# Harnessing the potential of long non-coding RNAs in breast cancer: from etiology to treatment resistance and clinical applications

**DOI:** 10.3389/fonc.2024.1337579

**Published:** 2024-03-05

**Authors:** Yun Wang, Na Bu, Xiao-fei Luan, Qian-qian Song, Ba-Fang Ma, Wenhui Hao, Jing-jing Yan, Li Wang, Xiao-ling Zheng, Yasen Maimaitiyiming

**Affiliations:** ^1^ Department of Pharmacy, Women’s Hospital, Zhejiang University School of Medicine, Hangzhou, China; ^2^ Department of Immunology, School of Basic Medical Sciences, Xinjiang Medical University, Urumqi, China; ^3^ Department of Biochemistry and Molecular Biology, School of Basic Medical Sciences, Xinjiang Medical University, Urumqi, Xinjiang, China; ^4^ Cancer Center, Zhejiang University School of Medicine, Hangzhou, China; ^5^ Women’s Hospital, Institute of Genetics, and Department of Environmental Medicine, Zhejiang University School of Medicine, Hangzhou, China; ^6^ State Key Laboratory of Pathogenesis, Prevention and Treatment of High Incidence Diseases in Central Asia, Xinjiang Medical University, Urumqi, China

**Keywords:** breast cancer, metastasis, therapy resistance, long non-coding RNA (LncRNA), competitive endogenous RNA (ceRNA), liquid biopsy

## Abstract

Breast cancer (BC) is the most common malignancy among women and a leading cause of cancer-related deaths of females worldwide. It is a complex and molecularly heterogeneous disease, with various subtypes that require different treatment strategies. Despite advances in high-resolution single-cell and multinomial technologies, distant metastasis and therapeutic resistance remain major challenges for BC treatment. Long non-coding RNAs (lncRNAs) are non-coding RNAs with more than 200 nucleotides in length. They act as competing endogenous RNAs (ceRNAs) to regulate post-transcriptional gene stability and modulate protein-protein, protein-DNA, and protein-RNA interactions to regulate various biological processes. Emerging evidence suggests that lncRNAs play essential roles in human cancers, including BC. In this review, we focus on the roles and mechanisms of lncRNAs in BC progression, metastasis, and treatment resistance, and discuss their potential value as therapeutic targets. Specifically, we summarize how lncRNAs are involved in the initiation and progression of BC, as well as their roles in metastasis and the development of therapeutic resistance. We also recapitulate the potential of lncRNAs as diagnostic biomarkers and discuss their potential use in personalized medicine. Finally, we provide lncRNA-based strategies to promote the prognosis of breast cancer patients in clinical settings, including the development of novel lncRNA-targeted therapies.

## 1 Introduction

Breast cancer (BC) may develop due to a variety of factors, including genetic mutations, lifestyle choices, and environmental exposures, with its incidence further influenced by various demographic and socioeconomic elements (1). Despite significant progress in cancer research frontier, BC remains a serious public health issue across the globe. According to latest statistics of GLOBOCAN, an estimated 2.3 million cases of BC were newly diagnosed from 185 countries, accounting for 11.7% of total cancer cases worldwide (1). BC is now recognized as the leading cause of cancer-related mortality in women, with 684,996 deaths reported in 2020. Predominantly attributed to rapid advancements in diagnostic technologies and an increase in the use of mammographic screening, BC has now surpassed lung cancer to become the most commonly diagnosed cancer in women globally. Importantly, it also ranks as the second most common malignancy worldwide, following closely behind lung cancer (1). While less common, BC does occur in men, making up approximately 1% of all BC instances globally (2). Hence, incessant research efforts are crucial to alleviate the significant public health, societal and economic burden posed by BC.

The incidence of BC is governed by a confluence of factors - genetic, epigenetic, and environmental, among others (3). In light of this, BC emerges as a multifaceted disease marked by vast heterogeneity in pathology, genomic alterations, gene expression profiles, and the tumor microenvironment (TME). BC is classified into different biological subtypes, with each subtype displaying unique pathological characteristics and diverse clinical outcomes (4). Indeed, while there have been substantial advancements in diagnostic techniques and treatment strategies over the past decade, the prognosis for BC patients remains unsatisfactory. Survival rates for BC are highly dependent on the stage at which the disease is identified, with early detection correlating to a higher survival advantage (5). Now, it is evident that metastasis accounts for the greatest proportion of BC-related mortality (6). Even though it is detected at an early stage, a notable percentage of women may see their disease evolve into a more aggressive subtype after undergoing initial therapy. This change is likely due to the molecular heterogeneity of BC. Molecular heterogeneity pertains to the genetic variation found within tumor cells, which can result in worse disease progression and resistance to treatment. This is why there is an ongoing focus on creating personalized and targeted therapies.

**Figure 1:**
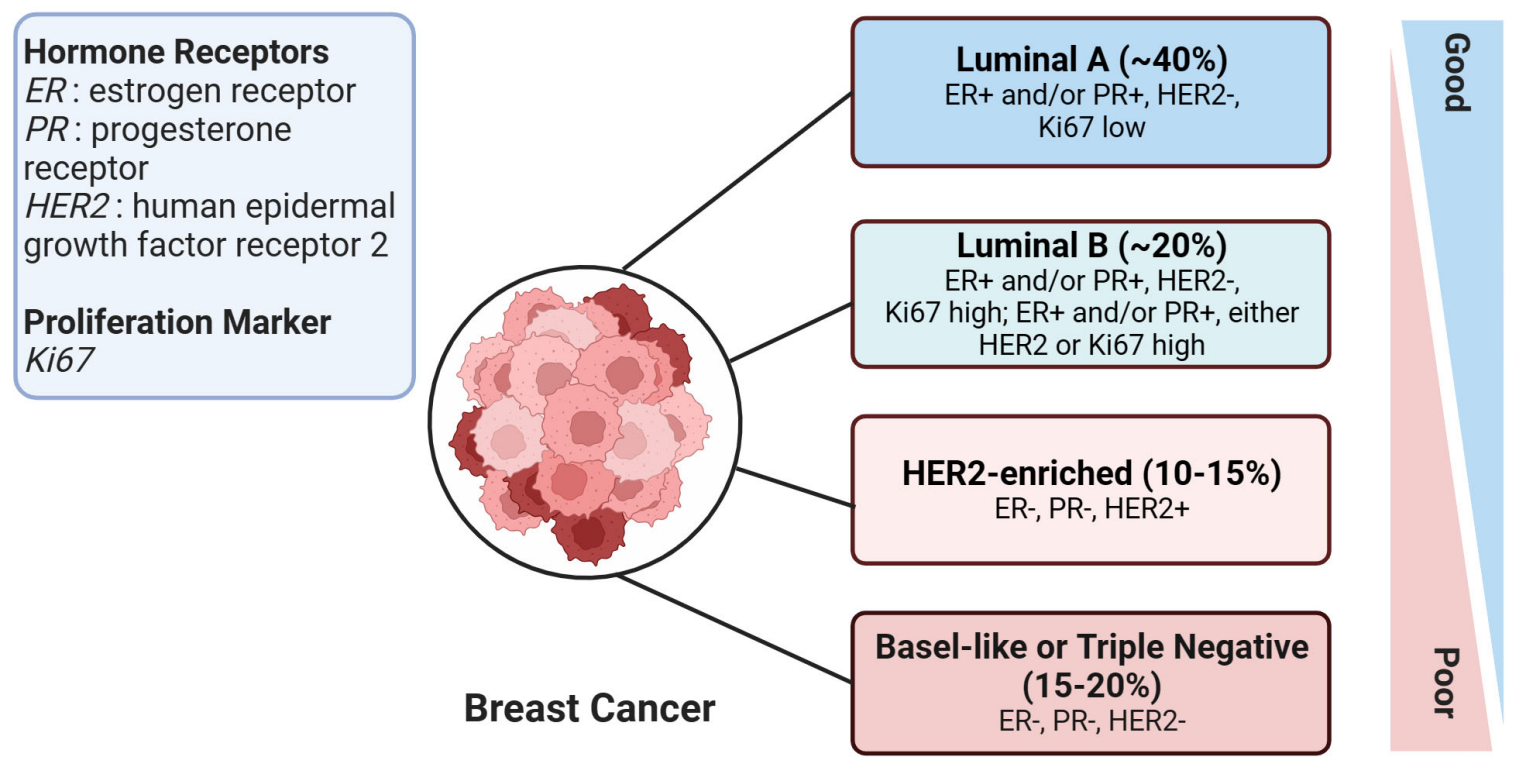
An overview of human breast cancer subtypes. This figure illustrates the various subtypes of breast cancer, with the approximate proportion (%) of each subtype among all breast cancer cases provided in brackets. Prognosis severity increases from top to bottom, signifying that the subtypes at the bottom are associated with worse prognoses. The figure was created in BioRender.com.

According to the St. Gallen guidelines, breast cancer (BC) is categorized into four subtypes (7). This categorization is based on the expression status of specific molecular biomarkers including the estrogen receptor (ER), progesterone receptor (PR), human epidermal growth factor receptor 2 (HER2), and Ki-67 labeling index, which is a marker of cell proliferation. The four subtypes of BC are Luminal A, Luminal B, HER2-enriched, and Basal-like subtypes (**Figure 1**). The Basal-like subtype is also known as triple-negative breast cancer (TNBC) due to the absence of ER, PR, and HER2 receptors, which makes this subtype particularly challenging to treat. With high metastatic properties and a lower rate of early detection, TNBC poses a significant challenge to treatment (8). Hence, improving survival rates in breast cancer patients, particularly those with the aggressive triple-negative subtype, necessitates the identification of novel molecular biomarkers. These biomarkers are key in assessing the risk of metastasis and treatment response, and additionally, there is a critical need to develop innovative therapies tailored towards tackling this formidable disease.

Rapidly evolving imaging techniques have prominently emerged as the primary tool for early diagnosis of breast cancer, supported by considerable clinical evidence suggesting their effectiveness in reducing breast cancer mortality (9). Further advancements in machine learning-based digital imaging techniques have notably enhanced diagnostic accuracy (10). However, their widespread application is curtailed by high costs and lack of specificity, rendering them unsuitable for early phase breast cancer detection (11). Similarly, conventional tumor diagnostic markers such as carcinoembryonic antigen (CEA), CA 15-3, and CA 125 prove impractical in detecting early phase breast cancer due to their low sensitivity.

Over the past few decades, there has been a continuous pursuit of diagnostic, prognostic, and predictive biomarkers to enhance breast cancer (BC) management. Numerous large molecules, such as DNA (APC, RARb_2_) (12), proteins (HER2,P53) (13), autoantibodies(MUC1) (14), and ncRNAs (non-coding RNAs), have been utilized as biomarkers for diagnosis. These biomarkers can be detected from serum samples, other body fluids, or tissues of tumor patients. Liquid biopsy, a more accessible, cost-effective, and repeatable sampling process, serves as a valuable source for such biomarkers, avoiding tumor heterogeneity issues (15). Long non-coding RNAs (lncRNAs) are non-protein-coding transcripts exceeding 200 nucleotides in length (16). Researchers have discovered abnormal lncRNA expression in both BC cell lines and tissues (17), revealing their potential role in breast cancer initiation and development. In this review, we synthesize the existing literature on lncRNAs’ roles in various aspects of BC and possible underlying mechanisms. This analysis offers novel insights into the diagnosis, prognosis, and prediction of BC, paving the way for improved management and treatment strategies.

## 2 Overview of ncRNAs

The advent of sequencing technologies has drastically revolutionized our understanding of the human genome. We now know that approximately 75% of the human genome is transcribed into RNA, yet only a small fraction— about 3%— translates into protein-coding mRNAs (18). The largest and most critical family of RNAs, non-coding RNAs (ncRNAs), do not code for any proteins. Despite their lack of protein-coding potential, ncRNAs fulfill vital roles in numerous pathophysiological processes, particularly in cancer. NcRNAs can be categorized into two main groups based on their size: long non-coding RNAs (lncRNAs) and small non-coding RNAs (sncRNAs). The latter is a collection of ncRNAs shorter than 200 nucleotides and includes microRNAs (miRNAs), PIWI-interacting RNAs (piRNAs), small interfering RNAs (siRNAs), small nucleolar RNAs (snoRNAs), and small nuclear RNAs (snRNAs) (19). In contrast, lncRNAs, as the name suggests, are longer than 200 nucleotides. Circular RNAs (circRNAs) constitute the third subgroup of ncRNAs. These distinguished by their covalent, close-loop (circular) single-stranded structures (20). Among these, lncRNAs represent the most intricate and challenging class of ncRNAs. These RNAs offer novel routes for research, potentially leading to new diagnoses, treatments, and understandings of diseases like cancer.

### 2.1 Classification of lncRNAs

LncRNAs were initially discovered as mRNA-like transcripts that possess both RNA- and protein-like functions. Generally, they lack significant open reading frames (ORFs) and are not translated into proteins, except for a few micropeptide-encoding lncRNAs (21). To date, over 27,000 lncRNAs have been annotated and the number continues to grow, while the functions of a large number of lncRNAs remain unexplored (22). Similar to mRNAs, most lncRNAs are transcribed by RNA polymerase II and are subjected to a series of processes including 5’-capping, splicing, and poly-adenylation at the 3’ end (23). LncRNA is the most complex type of ncRNA, with no uniform standard for classification. They can be categorized by length, genomic location, and mechanism of action (24). Based on their genomic localization, they can be classified as intronic, intergenic, sense, antisense, and enhancer lncRNAs. According to their mechanisms of action, they are categorized into four types: guide, scaffold, decoy, and signaling lncRNAs (25). Guide lncRNAs can bind to transcription factors and direct them to specific targets. Scaffold lncRNAs act as a “central platform” that binds different effector molecules simultaneously, like a “scaffold,” to integrate various signal pathways. Silencing guide and scaffold lncRNAs can result in altered localization or even loss of function of effector molecules. Decoy lncRNAs interact with target transcriptional regulators and block downstream signals. Signaling lncRNAs regulate downstream gene transcription without protein translation (26).

LncRNAs play a crucial role in the regulation of the genome and have a high level of tissue specificity, suggesting their integral role in maintaining cellular functions (21). They are involved in numerous biological processes, such as transcription, splicing, and translation. Notably, they can participate in chromatin remodeling and epigenetic regulation (16). Additionally, lncRNAs have been found to be dysregulated in various types of cancers, indicating that their abnormal expression or function could contribute to cancer development or progression (17). Thus, they hold promise as biomarkers for early detection, prognosis, and potential therapeutic targets in cancer treatment.

### 2.2 Subcellular localization and biological functions of lncRNAs

Though originally characterized as “transcriptional noise” without biological functionality, the biological activity and influence of lncRNAs on various pathophysiological processes have garnered increasing attention. The function of lncRNAs largely depends on their subcellular localization (23). These molecules are known to localize in both the nucleus and the cytoplasm (27). The majority of lncRNAs are found in the nucleus, the site of their biogenesis and processing, where they perform their functions. Nuclear lncRNAs are involved in gene regulation at both the epigenetic and transcriptional levels. They can bind directly to DNA or transcription factors and assist in the regulation of chromatin structure (28). Other lncRNAs require export to the cytoplasm where they target mRNAs, miRNAs, and proteins to regulate gene expression post-transcriptionally and translationally (29). For instance, lncRNAs can operate as “miRNA sponges” or as competitive endogenous RNA (ceRNA). These lncRNAs competitively bind with miRNAs, which allows them to indirectly control the expression of target genes at the post-transcriptional level. Moreover, recent research has begun to explore the subcellular localization of lncRNAs, shedding light on their presence in specific organelles such as mitochondria and the endoplasmic reticulum (ER) (30).

LncRNAs play crucial roles in various biological processes, including embryonic development, organogenesis, immune function, stem cell differentiation, and pluripotency, by regulating gene expression and protein translation (31). Notably, numerous lncRNAs display dysregulation in a variety of malignancies, with the up- or down-regulation of these lncRNAs either promoting or inhibiting tumor progression. Broadly speaking, lncRNAs can be divided into two categories: oncogenic and tumor-suppressive (32). Oncogenic lncRNAs, which typically exhibit overexpression in tumor tissues compared to healthy samples, play a role in promoting tumorigenesis. As a result, inhibiting these lncRNAs presents a promising potential anticancer strategy. Conversely, lncRNAs with tumor-suppressive properties are generally downregulated in cancerous tissues. Increasing the levels of these lncRNAs could serve as an approach for combating the disease. Moreover, lncRNAs have been found to contribute to tumor metastasis and therapeutic resistance (33). This makes them invaluable subjects of study in cancer research, as many could be developed into novel biomarkers for the diagnosis, progression, invasion, metastasis, and prognosis of cancer, or even as targets for therapeutic intervention.

### 2.3 Exosomal lncRNAs

Exosomes are tiny, nano-sized extracellular vesicles (EVs) that can be found in various human body fluids like blood, urine, and saliva (34). These exosomes can be secreted by all types of cells, and their formation begins when the plasma membrane invaginates to create intraluminal vesicles (ILVs), which then mature into multivesicular bodies (MVBs). These MVBs either fuse with the plasma membrane to release exosomes into the extracellular space or they get degraded in lysosomes (35). The contents of these exosomes can vary greatly, depending on the specific tissues and organs. They may contain nucleic acids (like mRNAs and ncRNAs), proteins, or even synthetic drugs, demonstrating that exosomes play a critical part in mediating communication between cells. The application of this knowledge extends further into cancer research, where it has been found that exosomes secreted by tumor cells contain tumor-specific long non-coding RNAs (lncRNAs), which reveal the original cellular pathophysiological state (36). These exosomal lncRNAs play a crucial role as regulators in the development of cancer. They are involved in various processes including the growth, proliferation, metastasis of cancer cells, promotion of angiogenesis, drug resistance, and immunomodulation, among other functions.

Indeed, changes in exosomal lncRNAs have been observed in various tumor types, suggesting that they can potentially serve as biomarkers for cancer diagnosis and prognosis (37). One of the advantages of exosomal lncRNAs is their high stability, which can be attributed to the protection provided by their lipid bilayers. This makes them more suitable candidates for developing biomarkers compared to other types of molecules. Moreover, unlike traditional *in-situ* biopsies, exosomes can be isolated from easily accessible body fluids like blood and urine, without the need for more invasive procedures. This makes the process of monitoring exosomes in body fluids much more convenient, feasible, and less invasive for patients (38). As a result, the utilization of exosomal lncRNAs as biomarkers holds great promise in advancing cancer detection and management.

### 2.4 LncRNAs-miRNAs-mRNAs network

**Figure 2:**
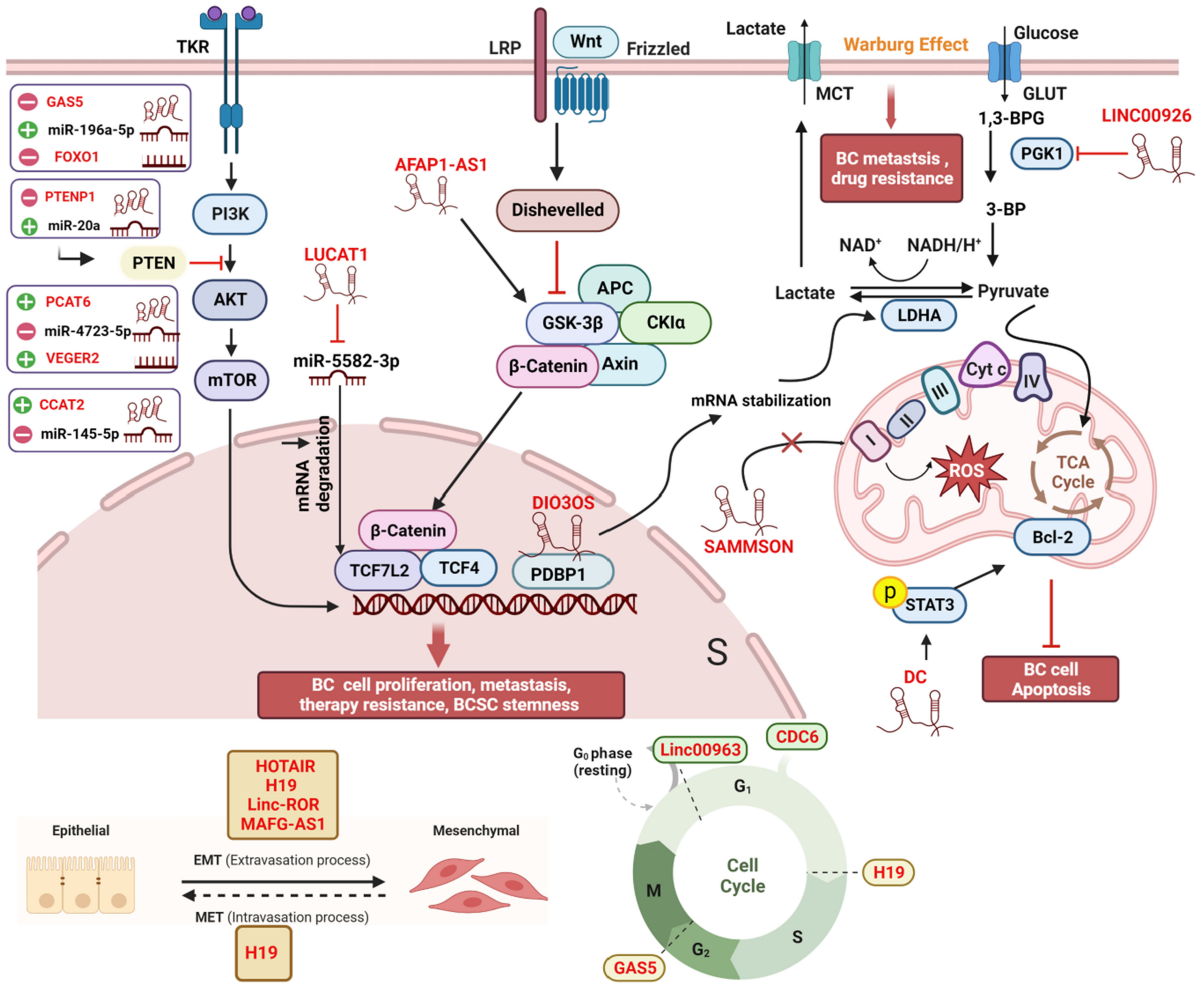
Comprehensive regulatory network of lncRNAs in breast cancer cells. Within the cellular environment, a complex regulatory network involving lncRNAs, their target genes, miRNAs, and interacting proteins is established. This network encompasses diverse signaling pathways, including Wnt/β-catenin, PI3K/AKT, and MAPK/ERK, coordinating various biological processes such as EMT, autophagy, the Warburg effect, oxidative phosphorylation, cell cycle arrest, angiogenesis, and treatment response in breast cancer patients. The lncRNA-miRNA-mRNA axis and ceRNA networks play pivotal roles in modulating gene expression during cancer development and progression, offering potential targets for therapeutic interventions and biomarker discovery. All lncRNAs are colored in red. “-” represents oncogene; “+” represents tumor suppressor; “┴“ represents inhibition; “↑” represents promotion. The figure was created in BioRender.com.

Micro RNAs (miRNAs) are small non-coding RNAs with lengths ranging from 17 to 25 nucleotides (39). Generally, miRNAs bind to the target mRNAs at their 3’untranslated regions (3’UTRs), subsequently inhibiting translation (40). A single miRNA can target multiple mRNA molecules, and a specific mRNA can be targeted by multiple miRNAs simultaneously. Like lncRNAs, aberrantly expressed miRNAs have been found in various cancer types, including BC (41). The mechanism of lncRNAs-mediated bioprocess regulation often involves miRNAs. In fact, lncRNAs interact with miRNAs through multiple mechanisms to regulate gene expression. Together, they form the lncRNAs-miRNAs-mRNAs axis. In other words, lncRNAs can act as “miRNA sponges” by competitively binding with miRNAs, indirectly regulating the expression of mRNAs (42, 43). These competing endogenous RNAs (ceRNAs) networks are extensively present in all aspects of breast cancer and are not listed separately in this article (**Figure 2**).

## 3 LncRNAs in breast cancer

Advancements in targeted therapy, immunotherapy, and innovative combination treatments have significantly enhanced the survival rate of patients with BC (44, 45). Nonetheless, BC still remains the leading cause of cancer-related mortality among women due to its high rates of recurrence, metastasis, and therapeutic resistance. One important reason for this is the complex and molecularly heterogeneous nature of BC. Until now, accumulating studies have demonstrated that lncRNAs play important roles in the progression, metastasis, and even treatment response of BC. Dysregulation of lncRNAs can disrupt normal transcription, leading to abnormal gene expression and ultimately tumor progression through various mechanisms (46). In both cancer and other diseases, a multitude of physiological and pathological processes are intricately linked to lncRNAs. Current studies have shown that the expression of lncRNAs in BC cells and tissues differs significantly from that in normal cells and tissues (47). A large number of lncRNAs have been shown to be involved in tumor progression, metastasis, as well as treatment resistance (**Figure 2**). Based on their functions and expression patterns in BC, lncRNAs can be classified as tumor suppressor genes or oncogenes, which will be discussed in detail below.

### 3.1 LncRNAs and BC progression

A large number of studies have provided evidence that lncRNAs are involved in BC progression, making them potential targets for biomarker design and discovery of novel anticancer drugs. In the following sections, we summarize several particular lncRNAs which play important roles in BC.

#### 3.1.1 Hotair

HOTAIR (HOX Transcript Antisense Intergenic RNA) is a long non-coding RNA (lncRNA) composed of 2158 nucleotides. It has the distinction of being the first lncRNA identified in correlation with a poor prognosis in breast cancer (BC). HOTAIR is considered an oncogenic lncRNA, and its overexpression has been reported in nearly all solid tumors (48). Notably, it is observed in both the cytoplasm and the nucleus. In terms of its mode of operation, HOTAIR acts as a “scaffold” that binds to and recruits the Polycomb Repressive Complex 2 (PRC2) to its target genes. Here, the histone methylase activity of PRC2 represses gene transcription (49). HOTAIR’s influence on BC is wide-ranging, contributing to the progression of BC, metastasis, and therapeutic resistance. Its overexpression has been identified in BC tissues and cells (50). In one study, Shi et al. found that HOTAIR enhances the proliferation, invasion, and migration of triple-negative BC cells through the HOTAIR/miR-203/CAV1 axis (51). Another study indicated that a complex of HBXIP, HOTAIR, and LSD1—where HOTAIR serves as a scaffold—can activate the pro-oncogenic transcription factor c-Myc, amplifying the growth of BC cells both *in vitro* and *in vivo* (52). Additionally, HOTAIR has been found to regulate the proliferation, migration, apoptosis, and invasion of MCF-7 cells by modulating the p53/Akt/JNK signaling pathway (53).

#### 3.1.2 H19

H19, the first identified long non-coding RNA (lncRNA) with riboregulatory function, is one of the most extensively studied lncRNAs in cancer (54). It is overexpressed in numerous solid tumors, including breast cancer (BC). H19 has been reported to bind with E2F1, a critical factor in the G1/S transition of the cell cycle. This association is linked to increased proliferation of BC cells as well as the progression to a more aggressive phenotype (55). As a miRNA sponge, H19 encourages BC cell proliferation, along with promoting invasiveness and migration. Conversely, the silencing of H19 can induce cell cycle arrest and apoptosis by regulating miR-138 and SOX4 (56).

#### 3.1.3 Other oncogenic lncRNAs

HOTAIR and H19 are classic and widely studied oncogenic lncRNAs in BC. In addition to these, numerous other lncRNAs have been identified to play oncogenic roles in BC progression. For instance, lncRNA CDC6 (cell division cycle 6) has been positively correlated with BC stages. Overexpression of CDC6 deregulates the G1 phase of the cell cycle, promoting BC cell migration. Concurrently, CDC6 acts as a molecular sponge for miR-215, further enhancing BC cell proliferation (57). NEAT1 (nuclear paraspeckle assembly transcript 1) is a structural component of nuclear paraspeckles (58). Although predominantly found in the nucleus, NEAT1 can be translocated to the cytoplasm, a process mediated by Pinin. In the cytoplasm, NEAT1 serves as a “scaffold” for the PGK1/PGAM1/ENO1 complex, promoting the penultimate step of glycolysis via substrate channeling (59). Accelerated glycolysis, also known as the Warburg effect, is a key metabolic change in cancer. Consequently, NEAT1 promotes tumor initiation, growth, and metastasis. GATA3 (GATA Binding Protein 3) is a transcription factor that regulates cell differentiation and acts as a tumor suppressor in BC progression. GATA3-AS1, the antisense RNA1 of GATA3, has been found to promote TNBC cell proliferation and migration by facilitating GATA3 degradation through ubiquitination (60). Additional lncRNAs related to BC initiation and progression are listed in **Table 1**.

**Table 1:** List of lncRNAs involved in BC progression.

LncRNA	Cellular localization	Subject	Mechanism of action	Biological functions
Oncogenic: up-regulated in BC cells and tissues				
ROR (61)	Nucleus	MCF-7	Acts as “decoy” of MLL1 to promote H3K4 methylation and increase TIMP3	Promotes BC cell proliferation and invasion, inhibits apoptosis
MAFG-AS1 (62)	Cytoplasm	MCF-7	MAFG-AS1/miR-150-5p/MYB axis	Enhances the BC cell viability and inhibits apoptosis
UCA1 (63)	Cytoplasm	MCF-7, MDA-MB-231	Up regulates PTP1B by sequestering miR-206	Promotes cell proliferation and colony formation *in vitro*
TDRKH-AS1 (64)	Not described	MCF-7, MDA-MB-231	miR-134-5p/CREB1 axis	Promotes BC cell proliferation and invasion
lncSNHG3 (65)	cytoplasm	T47D, MDA-MB-231, MDA-MB-468 etc	increasing CSNK2A1 expression level	Promotes malignant progression of breast cancer
AL133467.1 (66)	Not described	MCF-7, MDA-MB-231	Not described	Impedes BC cells’ proliferation and migration
Antitumor lncRNAs: down-regulated in BC cells and tissues.				
RP11-551L14.4 (67)	Not described	T47D, BT474	miR-4472	Inhibits cell proliferation, colony formation and attenuates cell cycle
MEG3 (68)	Cytoplasm	MDA-MB-231, MCF-7	MEG3/ miR-141-3p /RBMS3 axis	Inhibits xenograft growth, promotes apoptosis
EGOT (69)	Not described	BT549	Hedgehog pathway	Inhibits cell viability and migration
MEG3 (70)	nucleus and cytoplasm	MCF-7 and BT-474	miR-330/CNN1 axis	decreases cells in S stage and promotes apoptosis
HCG11 (71)	Not described	MCF7 and BT474	SRSF1/β-catenin axis	suppresses cell proliferation
LacRNA (72)	Cytoplasm	MDA-MB-231, BT549, T47D	stabilizes PHB2 and represses MYC targets	suppresses breast cancer metastasis
Dual-effect lncRNAs				
DANCR (ANCR) (73)	Cytoplasm	MDA-MB-231 MDA-MB-468	Recruits EZH2 to inhibit the transcription of SOCS3	Promotes cell viability and migration *in vitro*, as well as xenograft growth *in vivo*
DANCR (ANCR) (74)	Cytoplasm	MDA-MB-231, MCF-7	Promotes EZH2 degradation	Inhibits cell migration and invasion

#### 3.1.4 Antitumor lncRNAs

There are several lncRNAs with diverse roles in the progression of BC. Among them, GAS5 (Growth Arrest Specific 5), a tumor suppressor gene, was first isolated from mouse NIH3 cells. It has been observed that the presence of GAS5 is diminished in TNBC tissues, linked to an aggressive disease phenotype. Conversely, the overexpression of GAS5 within TNBC cells considerably promotes apoptosis (programmed cell death) in cancer cells while also inhibiting cell division (75). From a mechanistic standpoint, GAS5 functions as a ceRNA, countering miRNA-196a-5p, thus negating the protumor effects of the miRNA-196a-5p/FOXO1/PI3K/AKT pathway. GAS5’s anticancer effects involve multiple interactions with various miRNAs and proteins to encourage the apoptosis of BC cells via several pathway (76). PTCSC3 (Papillary Thyroid Carcinoma Susceptibility Candidate 3), found to be a tumor suppressor in numerous cancers, may serve as an upstream inhibitor of H19, regulating cell proliferation in TNBC cells. While it’s common for lncRNAs to modulate miRNAs, mRNAs, and chromatin, the regulation of one lncRNA by another is a seldom-witnessed phenomenon (77). PDCD4 (Programmed Cell Death 4) is a well-documented tumor suppressor gene. Its NATs (Natural Antisense Transcripts), known as PDAD4-AS1, can enhance the expression of PDCD4 through stabilizing PDCD4 RNA. Both PDCD4 and PDAD-AS1 negatively regulate BC cell proliferation by inhibiting cell cycle progression (78).

#### 3.1.5 Dual-effects lncRNAs

The above-mentioned lncRNAs have been shown to have either a promoting or inhibitory effect on the progression of BC. However, in certain circumstances, specific lncRNAs exhibit dual effects on BC progression. A single lncRNA can have opposite effects on the same subtype of BC or conflicting effects on different subtypes. For instance, MALAT1 (metastasis-associated lung adenocarcinoma transcript 1) has been identified as an oncogenic lncRNA that promotes the progression and metastasis of BC (79). In contrast, Kim et al. discovered that deficiency of MALAT1 induces BC metastasis, which can be reversed by the exogenous supplementation of MALAT1. This was observed in genetically engineered mouse models and xenograft models (80). Interestingly, another study found no differences in serum MALAT1 levels between BC patients and healthy controls (81). PTENP1 (phosphatase and tensin homolog pseudogene 1) is a pseudogene of the tumor suppressor PTEN, with a highly homologous region upstream of PTEN’s 3’-UTR. PTENP1 represses cell proliferation and promotes apoptosis. Gao et al. discovered that both PTENP1 and PTEN are downregulated in ER-positive cell lines MCF-7 and T47D. Overexpression of PTENP1 suppresses BC progression, while knockdown of PTENP1 enhances malignant behavior in these BC cells (82). Mechanistically, PTENP1 acts as an antitumor lncRNA by sponging miR-20a and regulating BC progression through the PTEN/PI3K/AKT pathway. Similar results were obtained in a previous study (83). However, another article mentions that upregulation of PTENP1 decreased PTEN gene expression in ER-positive MCF-7 and T47D cells and accelerated MCF-7 tumor growth *in vivo*. On the other hand, PTENP1 upregulation increased PTEN transcript levels and inhibited the growth rate of ER-negative MDA-MB-231 cells, suggesting that PTENP1 influences BC growth depending on the ER status (84).

XIST (X inactive-specific transcript), a key initiator of X chromosome inactivation in female mammals, has been recognized to play important roles in tumor progression regulation. Several studies have shown that XIST is downregulated and acts as an anti-cancer factor in BC. Knockdown of XIST promotes proliferation of MCF-7 cells and ovarian cancer cells. Mechanistically, XIST competes with miR-101 to upregulate C/EBP and KLF6 expression, which inhibits macrophage polarization toward the M2 phenotype, thereby suppressing BC cell proliferation and migration (85). Conversely, Zhao et al. found that XIST expression is upregulated in BC tissues and cell lines, and XIST knockdown significantly represses cell proliferation, migration, invasion, and anti-apoptotic activities in BC cells (86). Mechanistically, XIST acts as a sponge for miR-125b-5p, thereby upregulating the expression of the BC promoter NLRC5.

### 3.2 LncRNAs and BC metastasis

Metastasis, the most devastating stage of cancer progression, is responsible for the majority of cancer-related deaths. Many cancers, including BC, tend to metastasize preferentially to specific organs, a phenomenon known as organotropism (87). BC tends to metastasize to the brain, bones, lungs, and liver (87). Despite its significance, the process of metastasis is not fully understood, which has hindered the development of early predictive methods and effective treatment options for metastatic BC patients. To gain the ability to survive and metastasize, cancer cells often undergo a series of changes, including genetic and epigenetic alterations, as well as metabolic reprogramming (88). Research has indicated that cancer stem cells (CSCs), epithelial-mesenchymal transition (EMT), and autophagy are the three main mechanisms driving tumor metastasis (89). Within BC, there exists a small subpopulation of cells known as tumor-initiating cells (TICs) or breast cancer stem cells (BCSCs), which have the ability to generate daughter BCSCs (90). These daughter BCSCs possess the capability for unlimited proliferation through self-renewal and differentiation into BC cells. Only BCSCs have the potential to form recurrent or metastatic tumors (91). EMT is a dynamic process in which epithelial cells lose their polarity and intercellular cohesion, transforming into migratory mesenchymal cells. Although EMT is reversible, it provides cancer cells with increased motility and migration capabilities by breaking down intercellular bonds in the epithelial cells (92). EMT plays a crucial role in cancer progression and metastasis. Furthermore, EMT is closely intertwined with CSCs; for example, BCSCs can derive from human mammary epithelial cells through induction of EMT (93). Autophagy is a self-degradative process that can have a dual role in tumorigenesis. In the early stages of tumorigenesis, it exhibits anticancer effects, while in later stages, it contributes to tumor cell proliferation and survival, playing a fundamental role in tumor maintenance (42).

Interestingly, numerous lncRNAs have been identified to be involved in the aforementioned three mechanisms. For instance, HOTAIR is known to play a key role in the invasion, proliferation, colony formation, and self-renewal capacity of BCSCs by regulating SOX2 and NF-kB (94, 95). MiR-7, a metastasis-suppressing miRNA, inhibits both SETDB1 and the cellular EMT process in BCSCs. In MDA-MB231 cells and BC patients, HOTAIR functions by inhibiting miR-7 (96). Furthermore, HOTAIR can regulate autophagy, which is critical for BC cell survival, through its interactions with matrix metalloproteins (97). The functions and mechanisms of lncRNAs involved in these three mechanisms of tumor metastasis are summarized in **Table 2**.

**Table 2:** Role and mechanism of lncRNAs involved in three metastasis axes of BC.

LncRNA	Role and mechanism in		
	BCSCs	EMT	Autophagy
H19	Sponges let-7 and increases the expression of LIN28 to promote BCSCs maintenance (98)	Sponges miR-200b/c and let-7b differently, modulates the reversible shifts between epithelial and mesenchymal states (99)	Promotes autophagy and inhibits EMT via H19/Let-7/LIN28 pathway (100)
ROPM	Comprise ROPM/PLA2G16/lipid metabolism axis to maintain BCSCs properties (101)	Not investigated	Not investigated
ROR	Increases stemness of BCSCs, actives Wnt/β-catenin pathway (102)	Prevents the degradation of miR-205, induces EMT (103)	Inhibits Gem-induced autophagy by decreasing miR-34a (104)
MAFG-AS1	Not investigated	Regulates the EMT of BC *in vivo* by targeting the miR-150-5p/MYB axis (62)	Inhibits autophagy by sponging miR-3612 to elevate FKBP4 (105).
LUCAT1	Increases stemness of BCSCs via competitively binding miR-5582-3p with TCF7L2 and activating the Wnt/β-catenin pathway (106)	Not investigated	Not investigated
LINC00511	Targets miR-185-3p/E2F1 as ceRNA to enhance Nanog expression and facilitate BC cells stemness (107)	Not investigated	Not investigated

In addition to the three mechanisms mentioned above, there are other ways in which lncRNAs contribute to metastasis. For example, a novel lncRNA called LINC02273 forms a complex with hnRNPL and activates the oncogene AGR2, thus promoting BC metastasis (108). Cancer cells often undergo metabolic changes, such as increased glucose uptake and glycolysis, to satisfy the energy requirements for their malignant behavior (109). LINC00926 retards BC metastasis by inhibiting the PGK1-mediated Warburg effect, thereby reducing glucose uptake and lactate production (110). Hypoxia is also a hallmark of the tumor microenvironment (TME). In the hypoxic TME, the activation of the HIF (hypoxia-inducible factor) pathway promotes tumor progression and metastasis (111). For instance, HIF-2-induced lncRNA RAB11B-AS1 enhances angiogenic factors VEGFA and ANGPTL4 in hypoxic BC cells, leading to angiogenesis and metastasis (112). Additionally, lncRNA PCAT6 facilitates TNBC metastasis by sponging miR-4723-5p and binding to USP14, resulting in enhanced stability of VEGFR2 protein and activation of the Akt/mTOR pathway (113).

### 3.3 LncRNAs and therapeutic resistance of BC

In recent years, significant progress has been made in the development of various therapies for the management of BC. These therapies include (i) surgery, (ii) chemotherapy (especially for TNBC patients), (iii) trastuzumab (a HER2-specific monoclonal antibody for HER2-positive patients), (iv) endocrine therapy (e.g., tamoxifen) for ER-positive patients, (v) radiation therapy, and (vi) immunotherapy (114). Unfortunately, the emergence of treatment resistance often leads to metastasis and recurrence of BC, rendering it an incredibly challenging disease to treat. Extensive research has been conducted to understand the mechanisms underlying treatment resistance. The prevailing view is that BC is a stem cell disease, with BC stem cells (BCSCs) being the critical cells responsible for chemo-resistance and radio-resistance during BC therapy (115). Furthermore, the emerging field of lncRNA research has shown that lncRNAs play an essential role in treatment resistance through various molecular pathways, including increasing drug efflux, suppressing apoptosis, promoting BCSCs stemness, and acting as ceRNAs. As a result, lncRNAs have the potential to serve as biomarkers and promising targets to overcome drug/radiation resistance in BC patients.

#### 3.3.1 LncRNAs and chemotherapy resistance

Among the different treatment options for BC, chemotherapy is the most widely used in clinical settings as it can improve patients’ survival rates. Chemotherapy drugs commonly used for BC treatment include taxanes (paclitaxel and docetaxel), anthracyclines (doxorubicin and epirubicin), platinum drugs, and 5-Fluorouracil (5-FU). However, chemotherapy resistance remains a significant challenge in breast cancer treatment. Cancer cells become resistant to chemotherapy drugs, causing cancer growth and spread. Several factors contribute to chemotherapy resistance in breast cancer, such as tumor heterogeneity, genetic mutations, the tumor microenvironment, upregulated drug efflux pumps, metabolic reprogramming, and epigenetic changes, among others. For more details on the lncRNAs involved in chemotherapy resistance, please refer to **Table 3**.

**Table 3:** Roles and Mechanisms of lncRNAs in BC chemotherapy.

Drug	LncRNA	Role	Mechanism
Paclitaxel	FTH1P3 (116)	Inducing	FTH1P3 acts as a sponge of miR-206 and increases expression of ABCB1 to trigger paclitaxel resistance
	LINC00160 (117)	Inducing	LINC00160 up-regulates TFF3 by recruited C/EBPα to promote paclitaxel resistance of MCF-7 cells
	MAPT-AS1 (118)	Inducing	MAPT-AS1 upregulates MAPT and its protein TAU, which competes against paclitaxel at the microtubules
	EGOT (119)	Blocking	EGOT enhances autophagosome accumulation by increasing ITPR1 expression, thereby sensitizes BC cells to paclitaxel
Docetaxel	EPB41L4A-AS2 (120)	Blocking	EPB41L4A-AS2 promotes docetaxel sensitivity by activating ABCB1
	H19 (121)	Inducing	H19 targets and sustains PARP-1 activity to mediate the resistance of BC cells and patients
Doxorubicin	MALATI (79)	Inducing	MALAT1 targets miR-570-3P to decrease sensitivity of BC cells to doxorubicin
	SAMMSON (122)	Inducing	SAMMSON increases glycolysis and decreases mitochondrial respiration, leading to doxorubicin resistance
	SNHG10 (123)	Blocking	SNHG10 up-regulates miR-302b via promoting methylation, and enhances doxorubicin sensitivity of TNBC cells
Epirubicin	lnc005620 (124)	Inducing	lnc005620 upregulates ITGB1 and decreases the effects of epirubicin
	NONHSAT101069 (125)	Inducing	NONHSAT101069 promotes epirubicin resistance via NONHSAT101069/miR-129-5p/Twist1 axis in BC cells
Cisplatin	DANCR (126)	Inducing	DANCR upregulates KLF5 and induces the cisplatin resistance in TNBC patients by inhibiting p27
	HULC (127)	Inducing	HULC upregulates IGF1R and increases the expression of tumor stem cell markers to enhance cisplatin resistance
5-fluorouracil	CCAT2 (128)	Inducing	CCAT2 activates mTOR pathway to trigger 5-Fu resistance
	SNORD3A (129)	Blocking	Sponges miR185-5p and upregulates UMPS to increase 5-FU sensitivity

#### 3.3.2 LncRNAs and tamoxifen resistance

Estrogen receptor-positive (ER-positive) BC accounts for 75% of all BC cases, and ER therapy is crucial to inhibit estrogen-dependent tumor growth (130). ER therapy is the first-line adjuvant therapy for ER-positive BC patients and has been shown to reduce the recurrence and mortality whether chemotherapy is given concurrently (131). Aromatase, a rate-limiting enzyme that converts androgen to estrogen, is a vital target for aromatase inhibitors (AIs) such as tamoxifen, letrozole, and anastrozole. However, AI drug resistance persists in clinical practice, leading to tumor recurrence and metastasis (132). Numerous lncRNAs have been identified to be involved in AI drug resistance. Of particular importance is tamoxifen, one of the most commonly used AI drugs. For example, lncRNA DIO3OS interacts with PTBP1 to upregulate LDHA mRNA stability, activating glycolytic metabolism in tamoxifen-resistant BC cells and promoting ER-independent cell proliferation both *in vitro* and *in vivo* (133). Additionally, DILA1 upregulates the oncoprotein Cyclin D1 by inhibiting its phosphorylation and subsequent degradation. The upregulation of Cyclin D1 promotes BC cell proliferation and leads to tamoxifen resistance in both *in vivo* and *in vitro* settings (134). Furthermore, HOTAIR is highly expressed in tamoxifen-resistant breast cancer patients compared to newly diagnosed patients before tamoxifen treatment. The upregulation of HOTAIR activates the ER transcriptional program, resulting in increased BC cell proliferation and tamoxifen resistance (135). Overexpression of H19 in BC cells and tamoxifen-resistant BC cells activates autophagy through the H19/SAHH/DNMT3B axis, which contributes to tamoxifen resistance in BC cells (136). For more lncRNAs involved in tamoxifen resistance, please refer to **Table 4**.

**Table 4:** Role and Mechanisms of lncRNAs in BC tamoxifen resistance.

lncRNA	Role	Mechanism
SNHG6 (137)	Inducing	SNHG6 decrease tamoxifen sensitivity of BC cells by inhibiting miR-101and inducing EMT
LINP1 (138)	Inducing	LINP1 downregulates ER protein and attenuates estrogen response to trigger tamoxifen resistance
DSCAM-AS1 (139)	Inducing	DSCAM-AS1 promotes propagation of tamoxifen-resistant BC cells and inhibits apoptosis
HNF1A-AS1 (140)	Inducing	HNF1A-AS1 sponges miR-363 to promote SERTAD3 expression, stimulating tamoxifen resistance of BC cells
CCAT2 (141)	Inducing	CCAT2 sponges miR-145-5p, and activates PI3K/AKT/mTOR signaling pathway to promote tamoxifen resistance
BNAT1 (142)	Inducing	BNAT1 activates ERα signaling in tamoxifen resistant BC cells
BDNF-AS (143)	Inducing	BDNF-AS acts as scaffold of RNH1/TRIM21, abolishes RNH1-regulated mTOR mRNA decay to activate mTOR pathway
ATXN8OS (144)	Inducing	ATXN8OS activates VASP via sponge miR16-5p and promotes BC cell migration and metastasis
DC (145)	Inducing	DC promotes phosphorylation of STAT3 and upregulates Bcl-2 and Bcl-xL to reduce tamoxifen-induced apoptosis
LINC00894-002 (146)	Blocking	LINC00894-002 upregulates miR-200a-3p and miR-1b-1p, consequently inhibits TGF-β and oncogenic ZEB1
Uc.57 (147)	Blocking	Uc.57 inhibits BCL11A and its downstream PI3K/AKT and MAPK pathways
ADAMTS9-AS2 (148)	Blocking	ADAMTS9-AS2 upregulates PTEN by sponging miRNA-130a-5p and improves BC cells’ sensitivity to tamoxifen

#### 3.3.3 LncRNAs and trastuzumab resistance

In the past, HER2-positive breast cancer patients often had a poor prognosis. However, the discovery of trastuzumab, a recombinant humanized monoclonal antibody that targets the extracellular domain of HER2, has significantly improved the outcomes for these patients (149). Although other anti-HER2 agents like pertuzumab and lapatinib have been developed, trastuzumab remains the gold standard treatment. Unfortunately, the effectiveness of trastuzumab is limited by the emergence of drug resistance (150). Recent research has identified several long non-coding RNAs (lncRNAs) that are closely associated with trastuzumab resistance, including SNHG14, ATB, and AGAP2-AS1 (**Table 5**).

**Table 5:** Role and Mechanisms of lncRNAs in BC trastuzumab resistance.

lncRNA	Role	Mechanism
AGAP2-AS1 (151)	Inducing	AGAP2-AS1 upregulates CPT1 and induces FAO to cause resistance
SNHG7 (152)	Inducing	SNHG7 inhibits miR-186 to promote proliferation, apoptosis resistance, migration and EMT of BC cells
ATB (153)	Inducing	ATB sponges miR-200c, upregulates ZEB1 and ZNF-217 and thereby induces EMT to promote trastuzumab resistance
ZNF649-AS1 (154)	Inducing	ZNF649-AS1 binds to PTBP1 and promotes ATG5 transcription to induce autophagy and trastuzumab resistance
SNHG14 (155)	Inducing	SNHG14 promotes H3K27 acetylation and increases PABPC1, leading to activation of NRF2 pathways and BC cell survival
GAS5 (156)	Blocking	GAS5 downregulates miR-21, increases G2/M cell cycle arrest and DNA damage to increase BC cells radiosensitivity

#### 3.3.4 LncRNAs and radiotherapy resistance

Radiotherapy (RT) is commonly used as an adjuvant treatment after surgery for various types of breast cancer, including TNBC, metastatic BC, and advanced BC, as it has shown great benefits in reducing recurrence (157). The success of radiotherapy depends on the radiosensitivity of the tumor, which is influenced by factors such as cancer stem cells (CSCs), the tumor microenvironment, DNA repair, and gene expression (158). Unfortunately, some breast tumors develop resistance to radiation, leading to treatment failure and recurrence. Understanding the mechanisms of radiation resistance is crucial for improving the efficacy of radiotherapy. Numerous studies have found associations between specific lncRNAs and radiation resistance in breast cancer. For example, LINC00963 has been found to be upregulated in breast cancer tissues and correlated with aggressive tumor characteristics. Knockdown of LINC00963 has been shown to enhance DNA damage and oxidative stress, making breast cancer cells more sensitive to radiation (159). LINC00963 achieves this through its interactions with miR-324-3P, which normally inhibits the expression of ACK1, a driver of tumor progression. Further information on lncRNAs implicated in radiation resistance is summarized in **Table 6**. These findings highlight the importance of lncRNAs in mediating resistance to trastuzumab and radiotherapy in breast cancer. Further research is necessary to elucidate the underlying mechanisms and identify potential therapeutic targets to overcome drug and radiation resistance in breast cancer patients.

**Table 6:** Role and Mechanisms of lncRNAs in BC radiation resistance.

lncRNAs	Effect	Mechanism
DUXAP8 (160)	Inducing	DUXP8 activates the PI3K/AKT/mTOR and inhibits E-cadherin and RHOB via interaction with EZH2 to enhance the radiation resistance
HOTAIR (161)	Inducing	HOTAIR acts as a sponge of miR-449-5p, upregulates the expression of HSPA1A to enhance BC cells radiation resistance
HOTAIR (162)	Inducing	HOTAIR increases radiation resistance by inhibiting HOXD10 and the PI3K/AKT-BAD signaling pathway in BC cells
FGD5-AS1 (163)	Inducing	FGD5-AS1 acts as a sponge of miR-497-5p, up-regulates the expression of MACC1 to enhance BC cells radiation resistance
AFAP1-AS1 (164)	Inducing	AFAP1-AS1 activates the Wnt/*β*‐catenin pathway and them induces radiation resistance of TNBC
GAS5 (156)	Blocking	GAS5 sensitizes BC cells to radiation by inhibiting DNA repair and sponging miR-21
LINC00963-FOSB (165)	Inducing	mediating transcriptional activation of UBE3C to induce ubiquitination-dependent protein degradation of TP73

### 3.4 Circulating lncRNAs as biomarkers of BC

Accurate tumor biomarkers play a crucial role in diagnosing and predicting the prognosis for patients. Continuing efforts are being made to identify new biomarkers with high sensitivity and specificity. These biomarkers are valuable for evaluating tumor stage, metastasis risk, treatment response, and the development of new therapies (166). Circulating nucleic acids, including circulating RNAs such as lncRNAs, miRNAs, and piRNAs, have emerged as a promising class of potential biomarkers for improving tumor diagnosis. Compared to circulating DNAs, circulating RNAs offer higher specificity and sensitivity, garnering significant research attention (167). This review predominantly focuses on circulating lncRNAs as biomarkers for breast cancer, given their stability and abundance in the bloodstream, making them reliable cancer biomarkers (168). As indicated by previous findings on changes in breast cancer cells and tissues, there is a growing focus on circulating lncRNAs. Encouragingly, certain circulating lncRNAs can reflect cancer status, and changes in these lncRNAs are correlated with the degree of tumor progression and clinical features. Furthermore, some of these lncRNAs can identify specific subtypes of breast cancer, while a few others may serve as potential therapeutic agents or treatment targets (168).

For instance, HOTAIR has been found to be significantly elevated in the serum of breast cancer patients compared to healthy individuals, suggesting its potential as a diagnostic biomarker (169). One study indicated that HOTAIR exhibits a stronger diagnostic capability for breast cancer than CEA and CA 15-3, given its association with ER, Her-2, and lymph node metastasis (170). Notably, these researchers have observed a significant decrease in HOTAIR expression levels post-surgery. However, some studies have suggested the existence of potential technical errors in previous experimental results, warranting further investigation to determine whether HOTAIR could be adopted as a prognostic marker (171). Therefore, further studies are needed to assess whether HOTAIR could be adopted as a potential prognostic marker.

**Figure 3:**
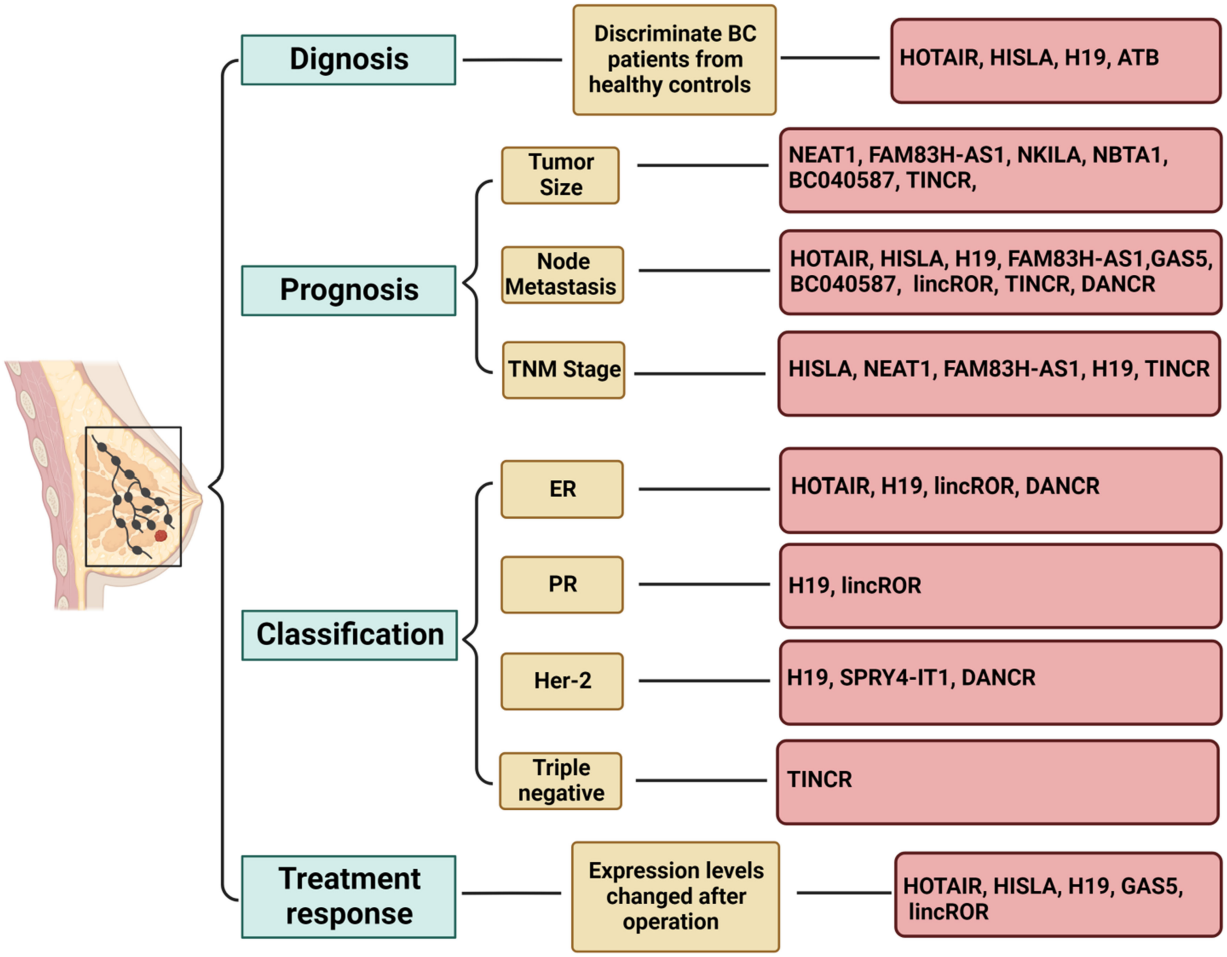
Role of circulating lncRNAs as potential biomarkers in breast cancer. Long non-coding RNAs (lncRNAs) have demonstrated potential as biomarkers in various aspects of breast cancer, including diagnosis, prognosis, subtype classification, and treatment response. Utilizing a combination of multiple lncRNAs may offer a viable strategy for leveraging lncRNA-based biomarkers in clinical applications. The figure was generated using BioRender.com.

Another example is HISLA (HIF-1α-stabilizing long noncoding RNA), which can be transmitted by extracellular vesicles from tumor-associated macrophages to breast cancer cells (172). HISLA is overexpressed in both breast cancer tissues and patient serum and is significantly associated with advanced grade, histological grade, distant metastasis, and poor survival. Moreover, serum levels of HISLA were observed to decrease significantly after surgery, suggesting its potential as a biomarker for diagnosing and prognosticating breast cancer (173). Similarly, H19 is released into the plasma from tumor cells upon breast cancer initiation, leading to an upregulation of plasma H19 levels. Consistently, plasma H19 levels were found to decrease significantly after surgery, and they have been significantly correlated with ER, PR, Her-2, and lymph node metastasis, indicating the potential use of H19 as a diagnostic and monitoring biomarker for breast cancer (174). In addition, a group of researchers detected the serum levels of lncRNAs PVT1, HOTAIR, NEAT1, and MALAT1 from Egyptian breast cancer and fibroadenoma patients, as well as healthy donors, and found that serum PVT1, HOTAIR, and NEAT1 could serve as potential biomarkers for breast cancer. Specifically, HOTAIR and NEAT1 demonstrated feasibility in differentiating between breast cancer and fibroadenoma (81). Moreover, El-Ashmawy et al. identified the upregulation of lncRNAs FAM83H-AS1 and ATB in the serum of breast cancer patients, with ATB exhibiting superior diagnostic accuracy compared to CA 15-3, a well-established serum protein marker (175). Conversely, FAM83H-AS1 demonstrated prognostic rather than diagnostic value, showing a significant association with tumor lymph node metastasis and tumor size (175). A brief overview of circulating lncRNAs with potential applications as biomarkers for breast cancer is presented in **Figure 3** (176–181). Some reported lncRNAs may exhibit relatively low specificity or sensitivity when used individually, making it feasible to combine several lncRNAs or use them in conjunction with traditional markers.

## 4 Therapeutic strategies against lncRNAs in breast cancer

In the ongoing battle against breast cancer, the significance of lncRNAs has come to the forefront of cancer research. Given their crucial role in the etiology and progression of breast cancer, as well as the development of resistance to current therapies, lncRNAs present an enticing target for novel treatments (182). Recent advances have led to the development of various strategies targeting lncRNAs, utilizing antisense oligonucleotides (ASOs), small molecules, and natural compounds.

ASOs represent one of the spearheads in the quest for lncRNA-targeted interventions. These synthetic molecules are engineered to be complementary to specific RNA sequences, allowing them to bind and neutralize lncRNAs through varied mechanisms. Among the mechanisms is the induction of RNase H-dependent degradation of the lncRNA, reducing its oncogenic influence in tumor cells (183). The potential of ASOs is illustrated by their effectiveness against HOTAIR and MALAT1, lncRNAs implicated in breast cancer metastasis and oncogenesis (184, 185). By subduing these lncRNAs, ASOs have demonstrated a capacity to inhibit cancer stem cell properties and reduce metastasis in preclinical models.

Small molecules like quercetin have also emerged as a viable approach to modulate lncRNAs. Quercetin, a widely available dietary flavonoid, has been found to diminish the expression of lncRNA MALAT1 in breast cancer cell lines (186). Through such downregulation, it exerts antitumor effects, likely by disrupting the complex interplay between key cellular pathways, p53, and miRNAs that revolve around MALAT1. This provides a compelling case for the therapeutic repurposing of naturally occurring compounds with regulatory effects on lncRNAs.

Additionally, natural products such as curcumin, recognized for its myriad health benefits, have been shown to regulate the expression of certain lncRNAs. A noteworthy example includes its ability to downregulate lncRNA H19, an action that can suppress the resistance of breast cancer cells to tamoxifen (187). Such findings underscore the potential therapeutic role of natural products in influencing lncRNA expression and combating drug resistance, critical hurdles in current treatment paradigms.

Collectively, the innovative application of ASOs, small molecules, peptides, and natural products holds immense promise for the future of breast cancer therapy, specifically through targeting lncRNAs. These emerging strategies may provide a breakthrough in addressing the challenges BC presents, including drug resistance, tumor recurrence, and metastasis, thereby improving outcomes for patients worldwide. As research progresses, the integration of these treatments into the clinical setting could transform the landscape of breast cancer management, offering hope for more effective and personalized therapeutic options.

## 5 Discussion and conclusion

Breast cancer (BC) remains a leading cause of cancer-related deaths in women, with different subtypes exhibiting distinct molecular characteristics and treatment responses. While significant progress has been made in improving the prognosis of BC patients through established treatments such as radical surgery and adjuvant therapy, a considerable number of patients still succumb to metastasis or resistance to these treatments. Therefore, there is a continuous need to explore novel therapeutic targets and molecular mechanisms.

The initiation and progression of breast cancer are influenced by a combination of genetic, epigenetic, and non-genetic factors. Previous research has primarily focused on genetic abnormalities and classic epigenetic factors such as histone and DNA modifications (188–191). Recently, ncRNAs, particularly lncRNAs, have emerged as crucial epigenetic regulators in cancer research (28). LncRNAs, which are longer than 200 nucleotides and have limited protein coding potential, play various biological roles in BC development through multiple mechanisms. Dysregulated lncRNAs have been closely associated with BC cell growth, apoptosis, invasion, EMT, autophagy, and therapeutic resistance, contributing to BC progression. Therefore, these lncRNAs hold promise as predictive biomarkers or therapeutic targets for BC patients.

In addition to downstream regulation, the upstream regulatory mechanism of lncRNAs has garnered significant research attention. One such mechanism involves N6-methyladenosine (m^6^A), the most frequent posttranscriptional modification found on mRNA, which has also been discovered on lncRNAs (192, 193). The interaction between m^6^A readers and lncRNAs, as well as the reciprocal regulation of m^6^A modifiers by lncRNAs, plays a role in BC development. For instance, the oncogenic lncRNA MIR210HG is induced by the m^6^A reader IGF2BP1 in an m^6^A-dependent manner to promote BC progression and metastasis (194). Another oncogenic lncRNA LNC942 bound to METTL14 (an m^6^A writer) directly and promoted m^6^A methylation and stabilization of CXCR4 and CYP1B1, promoting BC cells proliferation and inhibiting apoptosis (195). Collectively, m^6^A/lncRNAs axis enriches BC regulatory network and provides novel targets for BC prevention and management.

Interestingly, some lncRNAs have been found to encode short peptides that possess functional roles in cancer. For example, the lncRNA HOXB-AS3 encodes a conserved 53-amino acid peptide that suppresses colon cancer growth (196). Similarly, some peptides encoded by lncRNAs are involved in BC initiation and progression. For instance, the lncRNA LINC00908 contains a small open reading frame (ORF) encoding a 60-amino acid polypeptide named ASRPS, which acts as a small regulatory peptide of STAT3 (197). Downregulation of ASRPS was associated with poor clinical outcome, indicating that ASRPS is a potential antitumor peptide. Mechanistically, ASRPS directly binds to STAT3, inhibiting its phosphorylation and downstream VEGF expression, thereby suppressing TNBC angiogenesis (197). Additionally, the lncRNA CTD-2256P15.2 encodes a micropeptide called PACMP that regulates BC progression, multiple drug resistance, and ionizing radiation resistance by maintaining CtIP abundance and promoting PARP-1-dependent poly (ADP-ribosyl)ation (PARylation) through direct binding to DNA damage-induced poly (ADP-ribose) chains (198).

In summary, a substantial number of lncRNAs have been identified as oncogenic in BC, with their overexpression associated with aggressive disease and poor patient survival. Conversely, certain lncRNAs exhibit tumor-suppressive effects and are frequently downregulated in BC. Hence, targeting oncogenic lncRNAs while activating antitumor lncRNAs can provide unique opportunities in the battle against BC. Advances in multiomics sequencing technologies and bioinformatics tools are anticipated to uncover an increasing number of lncRNAs involved in BC progression, expanding the repertoire of diagnostic and prognostic biomarkers. With deeper understanding of the role of lncRNAs in BC initiation and progression, oncogenic lncRNAs may be developed as potential therapeutic targets for BC treatment. However, it is important to note that most studies investigating lncRNAs and BC are still at the preclinical stage, and further research is required to elucidate the underlying mechanisms. Future studies should focus on identifying specific and sensitive lncRNAs for early diagnosis, risk stratification, prognosis monitoring, and personalized treatment of BC.

## Author contributions

YW: Formal analysis, Investigation, Methodology, Software, Validation, Visualization, Writing – original draft. BN: Data curation, Investigation, Methodology, Software, Validation, Writing – original draft. XL: Conceptualization, Funding acquisition, Methodology, Resources, Writing – review & editing. QS: Investigation, Methodology, Software, Validation, Writing – original draft. B-FM: Investigation, Software, Visualization, Writing – original draft. WH: Investigation, Validation, Visualization, Writing – review & editing. JY: Investigation, Methodology, Visualization, Writing – original draft. LW: Conceptualization, Methodology, Visualization, Writing – review & editing. XZ: Methodology, Software, Visualization, Writing – review & editing. YM: Conceptualization, Data curation, Formal analysis, Funding acquisition, Investigation, Methodology, Project administration, Resources, Software, Supervision, Visualization, Writing – original draft, Writing – review & editing.
